# How to Organise Both

**Published:** 1918-08-17

**Authors:** 


### THE MATRONS' AND SISTERS' DEPARTMENT.

### THE ORGANISING OF THE NURSING AND DOMESTIC STAFF OF A HOSPITAL.

THERE is no more interesting problem for the matron than the organisation of the nursing department of a new hospital or the reorganisation of an old one. She should start by planning out the hours for the night and day staffs and then the off-duty hours for the day staff. She has to consider the number of beds for each sister, the number of nurses and probationers required, that of servants for the home and kitchen, wards and laundry, and the meal hours for each. When the matron has made out her rough scheme she should write in a note-book to be given to each worker a time-table of duties, meal hours, and hours on and off duty. Each note-book should be labelled, "Sister, Ward A.," "Pro. I., Ward A.," "Head Cook," "Scullery Maid I.," etc., with a note clearly inscribed on the cover that the book is to be kept clean and returned when the work is changed. In each sister's book should also be written the nurses' and servants' meal hours. The ward-probationer books should be made out after consultation with the sisters, and the matron should make the sisters understand that she would be glad to consider any improvements they can suggest after trying the scheme for a month.

In arranging the hours for the night staff, various points must be considered. Is it advisable to have the night and day staffs overlapping for a short time in the morning or not? Should the work be arranged so as to have the minimum number of nurses on night duty? Most authorities agree that it is best so to arrange as to have as few on night duty as possible, and that the hours of the night and day staffs should not overlap. Probably the best hours for the night staff are: Breakfast, 8.30 P.M.; on duty, 9 P.M.; midnight meals (out of the wards), 1 A.M. to 1.45 A.M.; off duty, 8 A.M. The night nurses should give the patients their breakfast at 6 A.M., see that any who can wash themselves have basins, etc., and see that the ward-maid does the sweeping thoroughly. The night nurses should have their dinner at 8.30 A.M., and be in bed by 1 o'clock. Great care should be taken that the night nurses have proper meals and that the midnight meal is well prepared. The night sister should have it impressed upon her that every nurse and probationer is to have three-quarters of an hour out of the wards. The hours for the day sisters might be from 8.30 A.M. to 9 P.M. Breakfast at 8 A.M.; lunch, 1 to 2; tea about 4; and dinner at 7; with three hours off duty daily. The third-year nurses' and probationers' hours would be 8 A.M. to 9 P.M. (9.10 for the third-year nurses, so as to have time to give the night nurse her report). Breakfast, 7.30 A.M.; on duty, 8; dinner, 12.30 or 1.15; tea, 4 to 4.30; supper, 7.30 to 8.

The washing of patients should be done by the day nurses, not the night ones, and by this means patients need not be awakened till breakfast or the 6 A.M. treatment is due. All these hours are arranged with the idea that the treatment hours are 10, 2, and 6.

The maids' hours might be arranged as follows: Breakfast, 6 A.M.; on duty, 6.30; morning lunch, 9; dinner, 12 and 12.30; tea, 4.30; supper, 8; with two or three hours off duty during the day. The night sister should visit the maids at their breakfast and take the roll-call. There should be night women to take on duty at 8 P.M. to 6 A.M. They would clear away the 7.30 and lay the 8 o'clock supper, see to the night nurses' breakfast and midnight meals, lay and cook the maids' breakfast, and in the night clean the night nurses' quarters.

The matron, or, in a large hospital, the assistant matron or home sister, should preside at the meals of the nursing staff and initial the report book. Absolute punctuality should be enforced at all meals, and every worker, from the junior scullery maid upwards, should be made to understand that she must keep closely to her time-table, because any deviation from it causes great inconvenience to a number of other workers.

Usually the best hours for the patients' meals are: Breakfast, 6 A.M.; lunch, 9.30; dinner, 12; tea, 4; and supper 6.30.

[*Criticisms and suggestions are invited and will be welcome.*—ED. THE HOSPITAL.]

### ROUND THE HOSPITALS.

